# Census Bulletin No. 9

**Published:** 1890-11

**Authors:** 


### BOOK NOTICES.

Census Bulletin No. 9. Robert P. Porter, Superintendent of the Census.

This bulletin announces the results of the census of the manufacture of pig-iron; from which it appears that the total production of pig-iron was 9,579,779 tons, whilst in the census year, 1880, it was 3,781,021 tons.

